# Metabolic engineering enables *Escherichia coli* to grow on 1,3-propanediol

**DOI:** 10.1016/j.synbio.2025.11.009

**Published:** 2025-12-22

**Authors:** Nga Yu Poon, Anthony J. Sinskey, Kang Zhou

**Affiliations:** a Department of Chemical and Biomolecular Engineering, National University of Singapore, Singapore; b Department of Biology, Massachusetts Institute of Technology, Cambridge, MA, USA; c Cluster of Food, Chemical and Biotechnology, Singapore Institute of Technology, Singapore

**Keywords:** Metabolic engineering, Synthetic pathway reconstruction, 1,3-PDO assimilation, Unconventional substrate utilization, Plastic waste upcycling, Adaptive laboratory evolution

## Abstract

1,3-propanediol (1,3-PDO) is used to synthesize plastics used in many consumer products. As the demand and production of such plastics increase, a technology will be needed to utilize 1,3-PDO released from the plastics after their disposal. In our previous study, we developed the strain (**BA07Δ**) that could use malonate semialdehyde (MSA, an important intermediate in the 1,3-PDO assimilation pathway) as the major carbon source. Here, we present construction of **PA16**, a strain which could grow to an OD_600_ of 7 by consuming 6.5 g/L of 1,3-PDO within 72 h in M9-based medium supplemented with 1 g/L of complete supplement mixture (CSM). This was achieved by adaptive laboratory evolution (ALE) after extending the pathway in **BA07Δ** through the introduction of a 1,3-propanediol dehydrogenase from *Klebsiella pneumoniae* (KpDhaT), an aldehyde dehydrogenase from *E. coli* (EcPuuC) and a 3-hydroxypropionate dehydrogenase from *Halomonas bluephagenesis* (HbDddA). Comparing the transcriptome of **PA16** and its ancestor in the ALE (**PA1**) revealed the upregulation of two genes, threonine dehydrogenase (EcTdh) and 2-amino-3-ketobutyrate CoA ligase (EcKbl) responsible for threonine degradation. The overexpression of these genes in **PA1** resulted in a 5-fold increase in the 72-h cell density. This finding helped simplify the growth medium of **PA16**: the supplement mixture containing more than 10 amino acids/nucleobases was reduced to just having 0.1 g/L threonine. **PA16**'s OD_600_ reached 3 when it grew in a defined medium containing 10 g/L 1,3-PDO and 0.1 g/L threonine as carbon sources. *E. coli***PA16** should be a useful strain to the subsequent research on upcycling 1,3-PDO derived from plastic wastes.

## Graphical abstract

**Figure:**
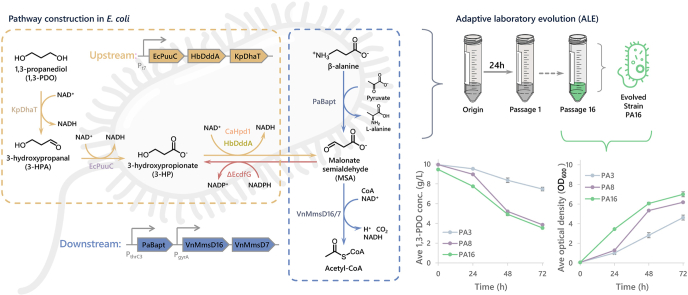

## 1 Introduction

Polytrimethylene terephthalate (PTT) is a semi-crystalline thermoplastic polymer known for its elasticity, good chemical resistance and low melting temperature. PTT is formed by polycondensation of intermediate esters made from 1,3-propandiol (1,3-PDO) and terephthalic acid (TPA). It exhibits properties that overlap with polyethylene terephthalate (PET) and polybutylene terephthalate (PBT) [1]. It is used by DuPont and Shell as a raw material to produce fabric Sorona® and Corterra® respectively [2,3]. PTT's quick-drying capability and resistance to scratching and wrinkle make it highly versatile, extending its use from home furnishings, such as carpets and pillowcases, to automotive upholstery.

The rise of PTT was partly driven by the increasing pressure on using renewable raw materials. Of the two components to produce PTT, 1,3-PDO could be produced from fermentation of glucose or glycerol with engineered bacteria [[4], [5], [6]]. DuPont Tate & Lyle BioProducts famously demonstrated the production of 1,3-PDO through *Escherichia coli* fermentation. *E. coli* was engineered to produce 1,3-PDO from glucose using genes from *Saccharomyces cerevisiae* and *Klebsiella pneumoniae* [7]. Following DuPont's success, other companies like METEX NØØVISTA (France) and Sheng Hong (China) also entered the market to produce bio-based 1,3-PDO production with engineered microbes from glycerol [8].

However, although PTT is classified as a Group 1 bioplastic, indicating that its production is fully or partially derived from biological sources, this does not imply that it is inherently biodegradable [9]. Effective strategies for the recycling and degradation of PTT remain critical aspects in assessing its long-term environmental sustainability. Some studies have demonstrated that PTT can be depolymerized into oligomers and monomers through processes including supercritical methanol or zinc acetate catalysed reactions [10,11]. In addition, hydrothermal treatment using hot compressed water at 240–320 °C has been shown to effectively hydrolyze PTT into its original monomers, 1,3-PDO and TPA [12]. Enzymatic depolymerization strategies initially developed for polyethylene terephthalate (PET) have also been reported to exhibit activity against PTT [13,14].

Of the two hydrolysis products, TPA can be readily recovered by acidic precipitation and filtration, whereas the recovery of 1,3-PDO from aqueous solutions remains costly due to its high polarity and elevated boiling point [15]. To our knowledge, no microbial strains have been developed that can directly utilize 1,3-PDO as the principal carbon source for biomass formation. This motivated us to expand *Escherichia coli*'s substrate scope to 1,3-PDO in this study.

To this end, we have chosen *E. coli* **MG1655** as a host bacterium for utilizing 1,3-PDO due to the availability of tools for genetic manipulation. In a previous study, we successfully constructed an *E. coli* strain which could utilize malonate semialdehyde (MSA, a hypothetic intermediate in 1,3-PDO degradation pathway) as a carbon source for *E. coli* growth. This was achieved by introducing two methylmalonate semialdehyde dehydrogenase from *Vibrio natriegens* (VnMmsDs) coupled with the disruption of a native 3-hydroxy acid dehydrogenase (EcYdfG) to prevent carbon loss through the formation of 3-hydroxypropionic acid (3-HP) from MSA forming the strain, **BA07Δ** [16].

**Fig. 1:**
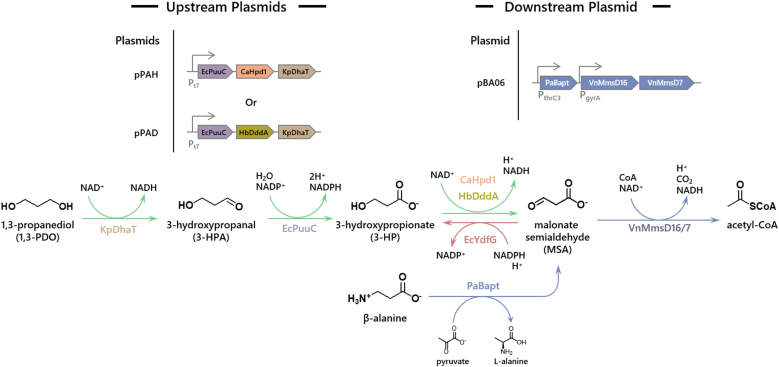
**An illustration of a possible 1,3-PDO assimilation pathway and the components of its construction.** The proposed metabolic pathway to extend the β-alanine assimilation pathway towards 1,3-PDO assimilation. The pathway is separated into up and down stream components, each controlled by a single plasmid. The downstream portion is controlled by plasmid pBA06 (in blue arrows). Two plasmid variants controlling the upstream portion (in green arrows), pPAH (**p**lasmid **P**ropanediol **A**ssimilating **H**pd1) and pPAD (**p**lasmid **P**ropanediol **A**ssimilating **D**daA) were tested. KpDhaT: *Klebsiella pneumoniae* 1,3-propanediol dehydrogenase; EcPuuC: *Escherichia coli* aldehyde dehydrogenase; HbDddA: *Halomonas bluphagenesis* 3-hydroxypropionate dehydrogenase; PaBapt: *Pseudomonas aeruginosa* β-alanine pyruvate transaminase; VnMMsDs: *Vibrio natriegens* Methyl malonate semi aldehyde dehydrogenases.

In this work, we have successfully constructed the full 1,3-PDO assimilating pathway in *E. coli* **MG1655** (Fig. 1). This was done by using **BA07Δ** as the platform for further engineering works, which included the introduction of a 1,3-propanediol dehydrogenase from *Klebsiella pneumoniae* (KpDhaT) [17], an aldehyde dehydrogenase from *E. coli* (EcPuuC) [18] and a 3-hydroxypropionate (3-HP) dehydrogenase from *Halomonas bluephagenesis* (HbDddA) [19]. The resulting strain was subsequently subjected to Adaptive Laboratory Evolution (ALE) to enhance the 1,3-PDO assimilation abilities. Our fastest-growing strain, **PA16** (**P**ropanediol **A**ssimilating passage **16**) was observed to consume 6.5 g/L of 1,3-PDO to achieve an OD600 of 7 within 72 h. **PA16** serves a chassis strain to enable future development of *E. coli* strains that can produce valuable chemicals from PTT wastes.

## 2 Results and discussion

### 2.1 Extension of the MSA assimilation pathway towards 1,3-PDO

To identify potential gene candidates to bridge the gap between 1,3-PDO and MSA, we surveyed the literature for potential candidates. A 3-hydroxypropionate (3-HP) dehydrogenase from *Candida albicans* (CaHpd1) was found to convert 3-HP into MSA [20]. The process is a defence mechanism against toxic propionyl-CoA accumulation. In another study, *Halomonas bluephagenesis* was used as a bacterial platform for the overproduction of 3-HP; A 3-HP dehydrogenase (HbDddA) was deleted as part of their strategy to prevent loss of 3-HP through MSA formation [19]. Thus, CaHpd1 and HbDddA became gene candidates for the conversion of 3-HP to MSA in our metabolic pathway.

Many genes from *Klebsiella pneumoniae* were used by DuPont in the construction of the *E. coli* strain for industrial production of bio-based 1,3-PDO. 1,3-PDO dehydrogenase (KpDhaT) was used to catalyze the last step to convert 3-HPA into 1,3-PDO. KpDhaT is also capable of catalysing the oxidation of 1,3-PDO into 3-HPA [17]. Finally, an aldehyde dehydrogenase (EcPuuC) from *E. coli*, capable of converting 3-HPA into 3-HP [18], was chosen to complete the metabolic pathway (Fig. 1).

Balancing expression levels of individual genes in a heterologous metabolic pathway is important in metabolic engineering, because some intermediates produced in the pathway may manifest certain stresses and undermine cellular integrity if left unchecked. Since the PDO-to-MSA segment involves production of a toxic aldehyde intermediate (3-hydroxypropanal, 3-HPA), we placed the aldehyde-utilizing gene (EcPuuC) at the first place in the operon when constructing the expression vector. It is assumed that the gene closest to the promoter of an operon has the highest expression level [[21], [22], [23]]. KpDhaT was positioned at the distal end of the operon to reduce its expression level. This arrangement left either HbDddA or CaHPD1 to occupy the middle position, giving rise to the construction of plasmids pPAD and pPAH, respectively.

### 2.2 Determining the efficacy of CaHPD1 and HbDddA through biotransformation

Our initial attempt of constructing the 1,3-PDO assimilating strain began with the introduction of pPAH into **BA07Δ**, forming the new strain, **BA07ΔH**. The newly constructed strain was subsequently cultivated overnight in LB for preparing the seed culture. Next, M9 minimal medium supplemented with 1 g/L of CSM and 5 g/L of 1,3-PDO (M9-CSM-5PDO) was inoculated with 1 % of the seed culture. Unfortunately, it was found that **BA07ΔH** could not grow on 1,3-PDO even after 7 days of cultivation.

We speculated that whole cell biocatalysis of **BA07ΔH** could be used to highlight potential issues with the pathway. If the pathway was impeded at any point, the accumulation of metabolic intermediates would occur due to the absence of branch points.

**Fig. 2:**
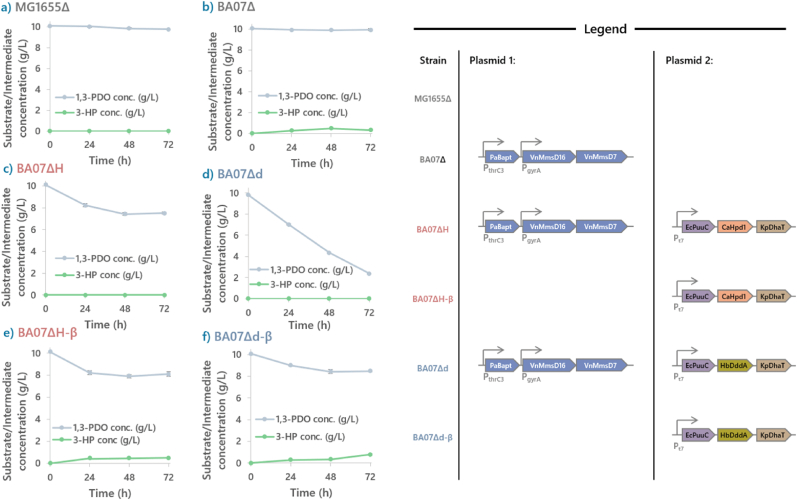
**Illustrations of 1,3-PDO consumption rates of whole cell biocatalysis of engineered strains.** In a progressive manner, the construction **BA07ΔD** began with the host strain **MG1655Δ**. Whole cell bio catalysis experiments was applied to test the significance of the upstream and downstream components of 10 g/L of 1,3-PDO. **(a)** and **(b)** illustrate the inability of **MG1655Δ** and **BA07Δ** to consume 1,3-PDO, suggesting the importance of the upstream pathway to enable 1,3-PDO consumption. **(c)** and **(d)** illustrate the 1,3-PDO assimilation profiles of **BA07ΔH** and **BA07ΔD**, where 2 g/L and 7.62 g/L of 1,3-PDO were consumed respectively, suggesting that HbDddA is the superior 3-hydroxypropionate dehydrogenase for our application. **(e)** and **(f)** show that without the downstream portion of the pathway, 1,3-PDO assimilation is greatly diminished.

The 1,3-PDO assimilation pathway is made up of the upstream portion, which is enabled by the plasmid pPAH (or pPAD), and the downstream portion is controlled by pBA06. To approach the investigation of the bottleneck in a systematic manner, these two plasmids were introduced sequentially into the host **MG1655Δ**. As a control, **MG1655Δ** was first tested to establish a baseline for 1,3-PDO assimilation. As expected, the 1,3-PDO assimilation rate of the host strain was low, having only consumed 0.88 g/L of 1,3-PDO, while producing 0.6 g/L of 3-HP within 72 h (Fig. 2a).

When **BA07Δ** (**MG1655 ΔEcYdfG** + pBA06) was subjected to the same tests, **BA07Δ** produced 0.4 g/L of 3-HP from 0.7 g/L of 1,3-PDO within 72 h (Fig. 2b), having similar 1,3-PDO assimilation profile as **MG1655Δ**. **BA07ΔH**, which harbours the complete 1,3-PDO assimilation pathway, consumed 3.8 g/L of 1,3-PDO within 72 h without any 3-HP accumulation.

Lastly, to establish the significance of the lower pathway, a strain devoid of the lower pathway was constructed (**BA07ΔH-β**). Unsurprisingly, 3-HP accumulation was observed: 1.6 g/L of 1,3-PDO was consumed and 1 g/L 3-HP was detected (Fig. 2e), consistent with the expected functionality of the lower pathway.

With the encouraging results from the biotransformation of **BA07ΔH**, the cells were taken from the 24-h time point of the biotransformation experiment and diluted tenfold using the M9-CSM-10PDO medium. Unfortunately, no growth was observed despite a 14-day incubation period.

*E. coli* **BA07ΔD** was subsequently constructed by introducing both pPAD and pBA06 into **MG1655Δ**. pPAD was the same to pPAH except that CaHPD1 was replaced by HbDddA. Under the same biotransformation conditions, **BA07ΔD** consumed 1,3-PDO at a rate twice as high as **BA07ΔH** (7.6 g/L vs. 3.7 g/L, Fig. 2c and d) without accumulating 3-HP. The efficacy of pPAD were also tested with the strain **MG1655ΔD-β**, where the downstream portion of the pathway was omitted. Seen in Fig. 2f, an accumulation of 0.79 g/L of 3-HP was observed.

A sample of **BA07ΔD** from the 24-h time point of biotransformation was diluted tenfold using fresh M9-CSM-10PDO medium. The culture was left at room temperature without agitation. Growth was detected 14 days later. The mutant strain (named as **PA0**) was found to have grown to an OD_600_ of 4.7 by consuming 3.8 g/L of 1,3-PDO. This was the first instance where *E. coli* was observed consuming 1,3-PDO as the primary carbon source for biomass formation.

### 2.3 Adaptive laboratory evolution of PA0

To enhance the 1,3-PDO assimilation phenotype of **PA0**, the culture underwent evolution through 16 serial passages. One passage involved inoculating fresh M9-CSM-10PDO medium with 1 % (v/v) of the previous culture. Here, the first passage was done with 1 % (v/v) of **PA0** culture from the 14th day, yielding the strain **PA1**. **PA1** grew to an OD_600_ of 4.2 by consuming 3 g/L of 1,3-PDO in 11 days. To test if the bacterium culture was contaminated with other species, the genetic material of **PA1** was subsequently extracted according to the protocol provided by the QIAGEN Plasmid mini kit and analyzed using Nanopore sequencing. Apart from the plasmid of interests, samples extracted from plasmid extraction kits also contains host genomic DNA, so the sequencing would acquire both genomic DNA and plasmid reads.

**Fig. 3:**
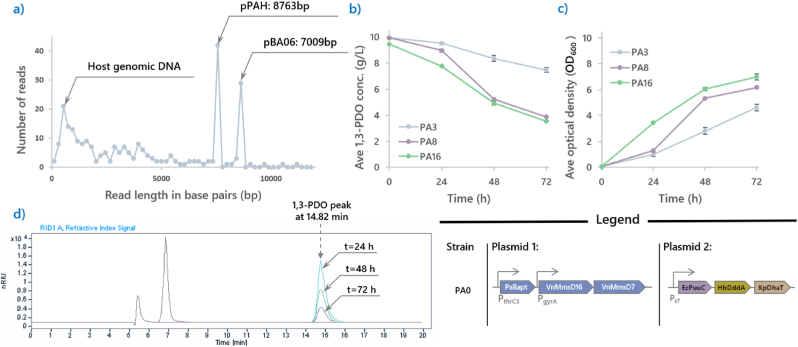
**Strain verification and ALE of strain PA0.** **(a)** Shows the representation of the frequency of each of individual read from the genetic material extracted from **PA1** as a plot diagram. It can be concluded that genetic material extracted from **PA1** comprise of the host genomic DNA which belongs to *E. coli***MG1655Δ** and pPAD and pBA06, proving that the mutant strain was not a contamination of a foreign bacteria species. **(b)** and **(c)** track the 1,3-PDO consumption and growth profiles of significant passages during the ALE of **PA0**. **PA16**, the fastest growing strain could consume 6.5 g/L of 1,3-PDO to achieve an OD_600_ of 7 within 72 h. **(d)** An overlay of chromatograms of the supernatant collected from culturing **PA16**. The two peaks on the left represents the salts present in all samples. The retention time of 1,3-PDO is 14.82 min as shown. The decline in concentration of 1,3-PDO across 72 h are shown here in blue (24 h), green (48 h) and purple (72 h). Also shown in the chromatogram, no intermediates were observed to be accumulated.

The species could then be identified by subjecting the raw reads from the genome to BLASTn to discern the identity of the mutant strain, while the plasmids could be aligned with existing plasmid maps created during their construction. From Fig. 3a, the genetic material extracted from **PA1** indeed consist of pPAD and pBA06, represented by the two peaks in the plot. BLASTn results of the genomic DNA also revealed that the sequences belong to *E. coli* **MG1655**.

In the next 15 serial passages (Fig. 3b–c), 1,3-PDO assimilation performance was greatly improved till the 8th passage (**PA8**). **PA8** consumed around 5 g/L of 1,3-PDO within 3 days (72 h), while the **PA0** was only able to assimilate 3.7 g/L in 14 days. It was observed that further improvements to 1,3-PDO assimilation after passage 8 was slowed, with **PA16** consuming 6.5 g/L of 1,3-PDO to achieve an OD_600_ of 7 within 72 h. As a control, **PA16** only grew to an OD_600_ of 0.6 when 1,3-PDO was not included in its growth medium. The PDO assimilation was oxygen-dependent. Under microaerobic condition (with culture tube sealed), the OD600 was only 1.4 at 72 h (Supplementary Fig. S1).

### 2.4 Transcription analysis of PA16

To understand the mechanism of 1,3-PDO assimilation, the RNA of **PA1** and **PA16** were extracted at the early exponential phase of their growth in M9-CSM medium supplemented with 10 g/L of 1,3-PDO. We hypothesize that the transcription fold changes between the least (**PA1**) and the most adapted strain (**PA16**) could yield useful insights into the biological conditions required of 1,3-PDO assimilation. There are two data sets which are of interest. The first is the expression levels of the genes belonging to the 1,3-PDO assimilation pathway. The second is the transcriptome of the host strain. Since the culture conditions, RNA extraction protocols and sequencing techniques are the same, the dataset between passages can be compared against each other [24].

**Fig. 4:**
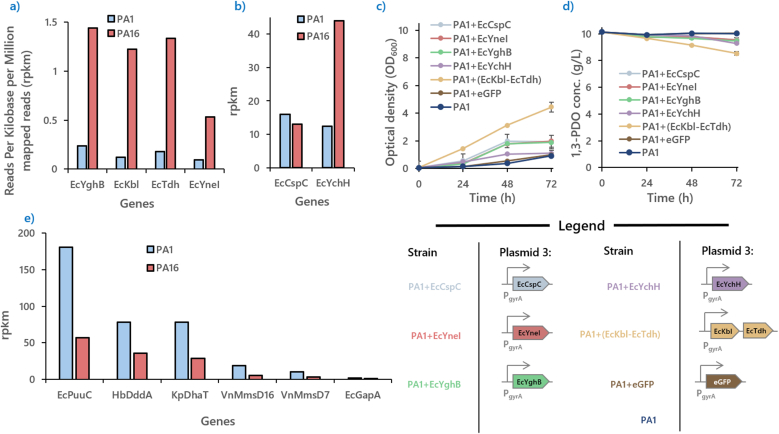
**Illustrations of expression levels of host genes essential to 1,3-PDO assimilation and the results of their subsequent overexpression.** **(a)** and **(b)** show gene candidates which are essential for 1,3-PDO assimilation was selected by comparing the transcriptome of **PA1** and **PA16**. The genes were subsequently introduced into **PA1** to improve 1,3-PDO assimilation. **(c)** and **(d)** shows their 1,3-PDO consumption profile and growth rates recorded over 72 h. It was observed that the upregulation of the EcKbl-EcTdh operon provided the most improvement leading to a 4.8-fold growth improvement in **PA1**. **(e)** Expression levels of plasmid-borne genes saw a huge decrease in expression levels between **PA1** and **PA16** with varying folds of reduction: EcPuuC at 2.6 folds; HbDddA at 1.8 folds; KpDhaT at 2.22 folds VnMmsD16 at 3 folds and VnmmsD7 at 2.7 folds. This suggests that lower expression levels of these genes helped **PA16** achieved better growth rates on 1,3-PDO. Since no mutation were detected on the replication origin nor promoter regions of the plasmids which could retard gene expression levels, it was speculated that the global transcription mechanism may be perturbed to lower expression levels of the genes. glyceraldehyde-3-phosphate dehydrogenase (EcGapA) is also shown here as a control comparison.

The expression levels of all the genes of the 1,3-PDO assimilating pathway were found to have been significantly reduced because of ALE (Fig. 4e). This was unexpected as it was originally speculated that since the genes for 1,3-PDO metabolism are essential for cellular survival on 1,3-PDO as the major carbon source, the genes would be upregulated instead.

However, it is important to note that although the expression levels of the 1,3-PDO assimilating genes in **PA16** have been downregulated, the expression levels from these genes were still among the highest expressed genes against with the transcriptomic background of the host. As a reference, glyceraldehyde-3-phosphate dehydrogenase (EcGapA), a key gene of glycolysis, were included in Fig. 4e for comparison.

This finding suggests that the initial expression levels of the genes may have been too high for the host cell to maintain, and that the ALE was used to reduce the expression levels of the genes in the 1,3-PDO assimilation pathway to a level where the stresses could be handled by the host defence mechanisms, enabling faster growth rates. Our initial suspicion was that the sequence of the plasmid promoters and replication origins were mutated to reduce gene expression rates and plasmid copy numbers respectively. However, this was not the case, as Nanopore sequencing suggests that the plasmid sequences remain unchanged.

An additional outcome of the transcriptomic analysis was the identification of host-derived transcripts with notable changes in abundance. These genes are stress-induced protein (EcYchH), stress protein (EcCspC), NAD(+)-dependent succinate semialdehyde dehydrogenase (EcYneI), inner membrane protein (EcYghB), 2-amino-3-ketobutyrate CoA ligase (EcKbl), threonine dehydrogenase (EcTdh) (Fig. 4a and b). The genes listed in Table 1 were prioritized based on their high transcription levels or significant fold changes (**PA16**/**PA1**). In this study, we focused on upregulated genes, since their functions can be easily tested through plasmid-based overexpression.

**Table 1:** List of genes of interest.

Gene	Product	PA1	PA16	PA16 VS PA1
EcYchH	stress-induced protein	12.48	43.92	3.52
EcCspC	stress protein, member of the CspA family	15.97	12.96	0.81
EcYneI	NAD(+)-dependent succinate semialdehyde dehydrogenase	0.10	0.53	5.53
EcYghB	Inner membrane protein	0.24	1.44	6.04
EcKbl	2-amino-3-ketobutyrate CoA ligase	0.12	1.23	10.20
EcTdh	threonine dehydrogenase	0.18	1.34	7.45
EcLpxC	UDP-3-*O*-acyl-N-acetylglucosamine deacetylase	2.67	11.89	4.45
EcCpxP	periplasmic protein	1.22	6.44	5.27
EcArgR	DNA-binding transcriptional dual regulator	0.80	3.02	3.77

Genes annotated as putative, transporters, and ribosomal proteins were not included in this table.

The following plasmids containing these candidates of interest were constructed and introduced into **PA1**: pPA-EcCspC, pPA-EcYneI, pPA-EcYghB, pPA-EcYchH and pPA-(EcKbl-EcTdh). EcKbl and EcTdh were amplified as a single fragment as they were found to be located adjacent to each other in the host genome. Unfortunately, attempts to construct plasmids carrying EcLpxC, EcCpxP, and EcArgR were unsuccessful, likely due to stress of overexpressing these genes. Each of the surviving strain was cultivated in M9-CSM-10PDO medium at 30 °C with 1,3-PDO concentrations and optical density tracked every 24 h for 72 h. Two controls were also included in this experiment: one was **PA1GFP**, which was created by using the same vector to express enhanced green fluorescence protein (eGFP) instead of candidate gene in **PA1**; the other was **PA1** without any further modifications.

As shown in Fig. 4c and d, it became evident that all the gene candidates introduced improved 1,3-PDO assimilation. Among the candidates, EcKbl and EcTdh were found to be the most helpful: **PA1**'s OD600 at 72 h was increased from 0.9 to 4.4 after the two genes were expressed. This strain (**PA1+**(**EcKbl-EcTdh**)) was also cultivated on M9-CSM without 1,3-PDO as a control, where no further growth was observed after 72 h.

**Fig. 5:**
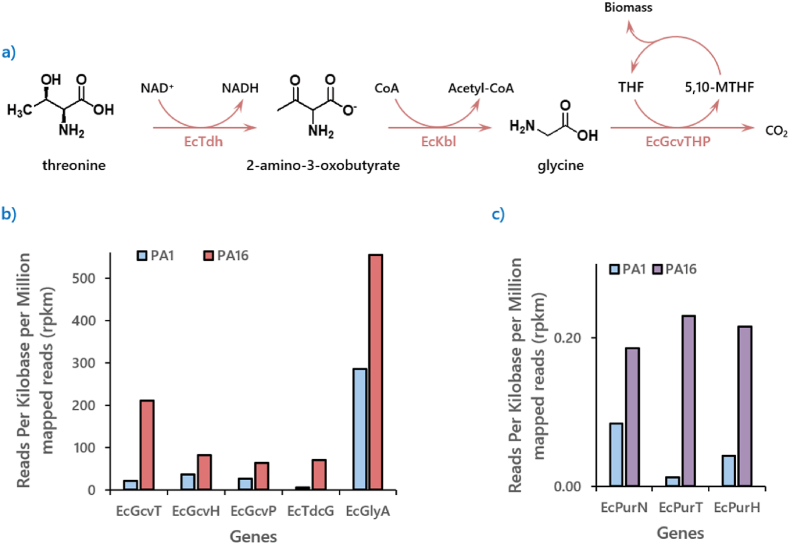
**Illustrates the potential connection between threonine degradation and C1 molecule supply.** **(a)** Illustrates the possible pathway for the conversion of threonine into pyruvate and 5,10-methylene-THF, both of which are critical intermediates for supporting biomass formation on 1,3-PDO. **(b)** Illustrates the great disparity of expression levels of the genes participating in the glycine cleavage system, along with two genes for converting glycine into serine (EcGlyA) and pyruvate (EcTdcG) between **PA16** and **PA1**. **(c)** Genes utilizing 5,10-methylene-THF as C1 building blocks for purine biosynthesis, essential for DNA synthesis were also discovered to have been upregulated in **PA16.**

EcKbl and EcTdh catalyze the two-step degradation of threonine into glycine, the latter of which can subsequently be cleaved into CO2 and a C1 methylene building block, which is required for the biosynthesis of nucleotides and amino acids (Fig. 5a). Consistent with this role, genes encoding components of the GCV were upregulated in **PA16** compared to **PA1** (Fig. 5b). Furthermore, genes involved in utilizing 5,10-methylene-THF for purine biosynthesis were also upregulated in **PA16** (Fig. 5c). We therefore hypothesize that the limited availability of C1 units in the 1,3-PDO assimilation pathway imposed an evolutionary pressure on **PA16** to enhance the expression of these pathways to support survival. We did not consider the contribution of additional acetyl-CoA here because acetyl-CoA is the entry point of 1,3-PDO assimilation flux into the central metabolism (acetyl-CoA should be abundant).

**Fig. 6:**
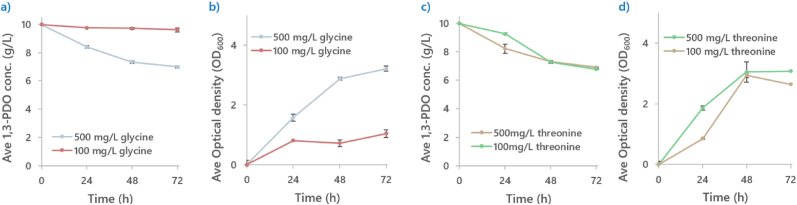
**Diagrams showing the performance of 1,3-PDO assimilation and growth profiles of P16 on 100** **mg/L or 500** **mg/L of glycine or threonine.** **(a-b)** Illustrates the 1,3-PDO and growth behaviour of **PA16** on 100 mg/L and 500 mg/L of glycine. Although growth rate and 1,3-PDO consumption was found to be minimal in 100 g/L of glycine, both parameters were improved where OD600 of 3.2 with 3 g/L of 1,3-PDO was consumed when glycine was increased to 500 mg/L after 72 h. **(c**–**d) PA16**'s growth and 1,3-PDO consumption was similar between the different concentrations of threonine, reaching an OD600 of ∼3 from ∼3.2 g/L of 1,3-PDO.

The above results highlighted the importance of threonine and glycine in supporting 1,3-PDO assimilation (1 g/L CSM contains 0.1 g/L threonine and no glycine). To test this hypothesis, glycine or threonine were supplemented at 0.1 g/L and 0.5 g/L. As shown in Fig. 6a and b, supplementation with 0.1 g/L of glycine resulted in minimal growth (OD_600_ ∼1 after 72 h) and poor substrate assimilation (0.4 g/L 1,3-PDO was consumed). Increasing glycine concentration to 0.5 g/L improved growth to an OD_600_ of 3.2 with 3 g/L of 1,3-PDO consumed. Supplementation with threonine at 0.1 g/L or 0.5 g/L yielded similar results, with **PA16** reaching an OD_600_ of ∼3 from ∼3.2 g/L of 1,3-PDO (Fig. 6c and d). The result confirmed the importance of threonine and glycine supplementation in 1,3-PDO assimilation and suggested that their benefiting effects may be related to supply of the C1 methylene building block to biomass formation.

### 2.5 Comparative genomic analysis of BA07Δ, PA1 and PA16

To further elucidate the genetic changes underlying 1,3-PDO assimilation, genomic DNA from **BA07Δ**, **PA1** and **PA16** was extracted and subjected to Illumina sequencing. As genome of the parental strain **BA07Δ** is not identical to the *E. coli* **MG1655** reference genome, **BA07Δ** was used as the baseline for comparative analysis against **PA1** and **PA16**. Among the mutations unique to the 1,3-PDO assimilating strains (**PA01** and **PA16**), notable changes were identified in a poly(A) polymerase I (EcPcnB), which adds poly(A) tails to RNA sequences, modulating RNA degradation [25]. In **PA1**, EcPcnB harboured a nonsynonymous mutation at residue 87, which changed from an alanine to glycine. In **PA16**, an additional mutation was observed at residue 31, substituting valine for leucine. These changes may have contributed to the reduced transcriptional stability of plasmid-borne genes observed in these strains as seen in Fig. 4e. Additionally, it was also reported that Rho-independent transcription terminators like the pheA terminator used in pPAD or T7 terminator in pBA06 serves as signals for polyadenylation and the subsequent degradation of the transcribed mRNAs [26]. These mutations in EcPcnB may be partially responsible for the reduced transcription level of the plasmid-based genes in the evolution from **PA1** to **PA16**.

### 2.6 Reconstruction of PA16

To further understand **PA16**, we extracted its plasmids pPAD and pBA06 and analyzed it using nanopore sequencing. We did not observe any mutation. As expected, these extracted plasmids could not enable the parent strain **BA07**D to grow on 1,3-PDO (in presence of CSM). This result suggests that spontaneous mutations in the genome essential for 1,3-PDO assimilation should have arisen during the evolution of **PA16**.

To test this hypothesis, we applied a plasmid curing strategy based on the Pfree system [27]. Briefly, a single colony of **PA16** was rendered competent and transformed with the Pfree plasmid, forming the strain **PA16-Pfree**. Following the published protocol, **PA16-Pfree** was cultured at 30 °C in the presence of rhamnose and anhydro tetracycline as inducers without antibiotics overnight. Subsequently, the liquid culture was streaked on antibiotic-free agar plates and incubated at 30 °C overnight. Individual colonies were screened for the loss of plasmid-born antibiotic resistance (spectinomycin and kanamycin) and for their ability to assimilate 1,3-PDO. The resulting plasmid-free derivative of **PA16** (designated here as **PA16Δpl**) lacked both resistance markers and the 1,3-PDO assimilation phenotype.

**Fig. 7:**
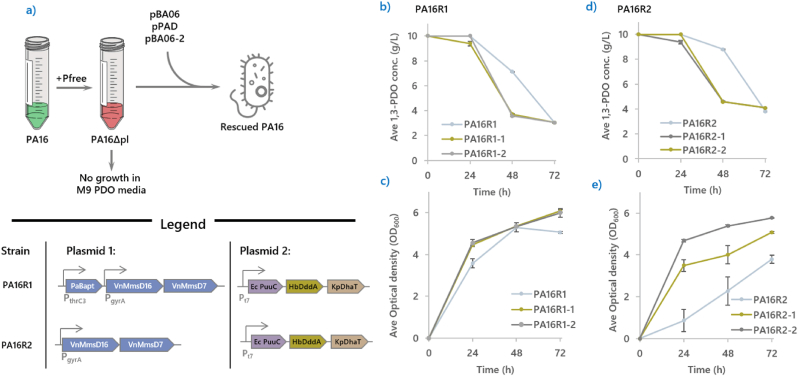
**Illustrations describing the reconstruction of PA16.** **(a)** A simple diagram illustrating the use of pfree system to cure **PA16** of pBA06 and pPAD. Upon successful curing, the new strain, **PA16Δpl** lost the ability to consume 1,3-PDO as the main carbon source. 1,3-PDO assimilation was restored when either pBA06 or pBA06-2 along with pPAD were reintroduced into **PA16Δpl**. **(b)** Shows the successful restoration of 1,3-PDO assimilation of **PA16R1**, and **(c)** shows the optical density of **PA16 R1**, where both parameters were improved after two passages. **(d)** and **(e)** illustrates the rate of 1,3-PDO assimilation and optical density of **PA16R2**, where both parameters were also improved after two passages.

To test if plasmid complementation could restore the phenotype, **PA16Δpl** was transformed with pBA06 and pPAD, forming the regenerated strain **PA16R1**. This strain regained the ability to assimilate 1,3-PDO (Fig. 7b and c) and, unlike its predecessors **PA1** or **PA0**, did not require prolonged incubation times of weeks to establish growth. **PA16R1** reached an OD_600_ of 5.06 by consuming 7 g/L of 1,3-PDO. Performance was further improved after two successive passages, which both **PA16R1-1** and **PA16R1-2** achieving and OD_600_ of 6 under the same conditions.

In parallel, **PA16Δpl** was also complemented with a modified pBA06 lacking PaBapt (pBA06-2), forming **PA16R2**. This strain initially grew to an OD_600_ of 3.79 from 6.2 g/L of 1,3-PDO, and after two passages, **PA16R2-2** achieved an OD_600_ of 5.7 from 6 g/L of 1,3-PDO (Fig. 7d and e). PaBapt is a legacy gene left in pBA06 from a previous study of β-alanine assimilation. The result confirmed that PaBapt is not needed for 1,3-PDO assimilation.

## 3 Materials and method

### 3.1 Chemicals and media formulation

All chemicals involved in this study were procured from Sigma Aldrich or from BioBasic. Cultivation of strains for plasmid propagation was done using LB. For metabolite analysis purposes, M9 minimal medium was used. The composition of the base M9 medium (final concentration) was as follows: 6.8 g/L Na_2_HPO_4_, 3 g/L KH_2_PO_4_, 0.5 g/L NaCl and 1 g/L NH_4_Cl.

For every 10 mL of M9 media, 17 μL of K3 master mixture was supplemented. The K3 master mix was prepared by mixing 2.5 mL of 0.1 M ferric citrate solution (autoclaved), 1 mL of 4.5 g/L thiamine solution, 3 mL of 4 mM Na_2_MoO_4_ (autoclaved), 1 mL of 1 M MgSO_4_ solution (autoclaved) and 1 mL of 1,000X K3 trace elements stock. The 1,000X K3 trace elements stock contained 5 g/L CaCl_2_∙2H_2_O, 1.6 g/L MnCl_2_∙4H_2_O, 0.38 g/L CuCl_2_∙2H_2_O, 0.5 g/L CoCl_2_∙6H_2_O, 0.94 g/L ZnCl_2_, 0.03 g/L H_3_BO_3_, 0.4 g/L Na_2_EDTA∙2H_2_O. The pH of the medium was adjusted to 7.

10 g/L 1,3-PDO and 0.8 g/L Complete Supplement Mixture (CSM) were supplemented in most M9 media. Such M9 medium is labeled as M9-CSM-10PDO. 0.8 g/L CSM is composed of Adenine hemisulfate (10 mg/L), l-Arginine (50 mg/L), l-Aspartic acid (80 mg/L), l-Histidine hydrochloride monohydrate (20 mg/L), l-Isoleucine (50 mg/L), l-Leucine (100 mg/L), l-Lysine hydrochloride (50 mg/L), l-Methionine (20 mg/L), l-Phenylalanine (50 mg/L), l-Threonine (100 mg/L), L-Tryptophan (50 mg/L), l-Tyrosine (50 mg/L), l-Valine (140 mg/L), Uracil (20 mg/L).

Solid media were prepared by adding 1.5 % (w/v) agar.

### 3.2 Bacterial culture

All strains cultivated in liquid media were done in 150 mL shake flask or in 50 mL Falcon tubes and were incubated at 30 °C/220 rpm. Liquid cultures of genetically modified organisms were supplemented with either 50 mg/L of Kanamycin and/or Spectinomycin to maintain plasmid of the modified organism. In the case of plasmid propagation and bacterial colony cultivation on solid culture, agar plates supplemented with either 50 mg/L of Kanamycin and/or Spectinomycin were incubated at 30 °C until obvious colonies could be observed. The colonies were picked for further processing.

ALE was performed in a series of passages. A passage is defined as the following: a first culture was cultivated for 24 h in 5 mL of M9-CSM-10PDO medium in a 50 mL Falcon tube; 50 μL of the grown culture was used to inoculate a fresh batch of 5 mL M9-CSM-10PDO medium.

### 3.3 Biotransformation

Biotransformation experiments begin with an overnight culture of engineered cells cultivated in LB. The cells were then pelleted, and the LB supernatant was discarded. The pellet was resuspended with 5 mL of M9-CSM-10PDO. The resuspended culture was incubated at 30 °C/220 rpm. 1,3-PDO concentration was monitored over time.

### 3.4 Metabolite analysis and optical density analysis

At a sampling time point, 200 μL of cell culture was collected for analysis. The samples were pelleted via centrifugation at 10,000 rpm for 1 min, and the supernatant was filtered with a 0.22 μm nylon filter. The filtrate was analyzed with an Agilent 1260 Infinity HPLC equipped with an Aminex HPX-87H column (300 mm × 7.8 mm, Bio-Rad) and Refractive Index Detector (RID). The mobile phase used in the analysis was 5 mM sulphuric acid aqueous solution, at a flow rate of 0.7 mL/min in isocratic mode. The column temperature was set at 50 °C.

A separate sample of 10 μL of cell culture was also collected from each time point for the purpose of cell density analysis. The sample was mixed with 190 μL of fresh LB or M9 minimal medium in a flat bottom 96 well plate (Costar). The Optical Density at 600 nm (OD_600_) was measured with a plate reader (Thermofisher). The obtained values were converted into the standard units by using a calibration curve.

### 3.5 Plasmid construction, sequencing and strain engineering

Newly constructed plasmids were propagated in Dh5α (NEB C2987H) overnight and extracted in accordance with the protocol provided in the QIAprep Spin Miniprep Kit (Qiagen). 50 ng of the extracted genetic materials were analyzed using the nanopore sequencing workflow provided by Oxford Nanopore Technologies with Flongle Flow Cell (FLG-114) and Rapid barcoding kit (SQK-RBK114). The analysis of the sequencing output was done with our in-house developed MATLAB application. The list of plasmids and strains used in this study can be found in Table 2, Table 3 respectively.

**Table 2:** List of plasmids constructed in the study of 1,3-PDO assimilation.

Plasmid name	Plasmid details
pPAH	P15(Kan^R^)T7: EcPuuC - CaHPD1 - KpDhaT
pPAD	P15(Kan^R^)T7: EcPuuC - HbDddA - KpDhaT
pCspC	P5(Cam^R^)gyrA: EcCspC
pYneI	P5(Cam^R^)gyrA: EcYneI
pYghB	P5(Cam^R^)gyrA: EcYghB
pYchH	P5(Cam^R^)gyrA: EcYchH
pEcKbl-EcTdh	P5(Cam^R^)gyrA: EcKbl - EcTdh
peGFP	P5(Cam^R^)gyrA: eGFP
pBA06-2	P5(Cam^R^)gyrA: VnMmsD16 - VnMmsD7

Kan^R^: Kanamycin resistance; EcPuuC: *Escherichia coli* β-alanine pyruvate transaminase; CaHPD1: *Candida albicans* 3-hydroxypropionate dehydrogenase; KpDhaT: *Klebsiella pneumoniae* 1,3-propanediol oxidoreductase; HbDddA: *Halomonas bluephagenesis* 3-hydroxypropionate dehydrogenase; EcYneI: *Escherichia coli* Succinic semialdehyde dehydrogenase; EcCspC: *Escherichia coli* cold shock protein C; EcYghB: *Escherichia coli* dedA-like inner membrane protein; EcYchH: *Escherichia coli* stress-induced protein; EcKbl: *Escherichia coli* 2-amino-3-ketobutyrate CoA ligase; EcTdh: *Escherichia coli* threonine dehydrogenase; eGFP: enhanced green fluorescence protein; VnMmsD: *Vibrio natriegens* methylmalonate semialdehyde dehydrogenase.

**Table 3:** List of strains constructed in the study of 1,3-PDO assimilation.

Strain name	Host strain	Plasmid 1	Plasmid 2	Plasmid 3
BA07Δ	MG1655ΔEcYdfG	pBA06	–	–
BA07ΔH	MG1655ΔEcYdfG	pBA06	pPAH	–
BA07ΔH-β	MG1655ΔEcYdfG	–	pPAH	–
BA07ΔD	MG1655ΔEcYdfG	pBA06	pPAD	–
BA07ΔD-β	MG1655ΔEcYdfG	–	pPAD	–
PA1+EcCspC	MG1655ΔEcYdfG	pBA06	pPAD	pPA-EcCspC
PA1+EcYneI	MG1655ΔEcYdfG	pBA06	pPAD	pPA-EcYneI
PA1+EcYghB	MG1655ΔEcYdfG	pBA06	pPAD	pPA-EcYghB
PA1+EcYchH	MG1655ΔEcYdfG	pBA06	pPAD	pPA-EcYchH
PA1+(EcKbl-EcTdh)	MG1655ΔEcYdfG	pBA06	pPAD	pPA-EcKbl-EcTdh
PA1+eGFP	MG1655ΔEcYdfG	pBA06	pPAD	pPA-eGFP
PA16+pfree	MG1655ΔEcYdfG	pBA06	pPAD	pfree
PA16pl	MG1655ΔEcYdfG			
PA16R1	MG1655ΔEcYdfG	pBA06	pPAD	
PA16R2	MG1655ΔEcYdfG	pBA06-2	pPAD	

**MG1655Δ** (ΔrecA, ΔendA, ΔydfG, DE3) was selected as the default host for plasmid transformation. Here, the ‘Δ’ designation denotes that the EcYdfG gene in **MG1655** was additionally disrupted.

### 3.6 RNA extraction and transcriptome analysis

**PA1** and **PA16** were grown on M9-CSM-10PDO. Total RNA was extracted during the early exponential phase denoted when OD_600_ was measured to be around 0.5 to 1, according to the manufacturer's protocol (Thermo Scientific GeneJET RNA purification kit, K0731). After verification of RNA concentrations using Nanodrop, the samples were sequenced using the Illumina platform through a third-party service provider (NovogeneAIT Genomics Singapore). The default workflow was used except that the rRNA depletion was omitted for cost-saving. The resulting FASTQ data was subsequently process with an in-house developed MATLAB application. The resulting transcription levels (in terms of rpkm values) from both samples were exported to an Excel spreadsheet file for differential analysis.

### 3.7 Genome extraction and analysis

**PA1** and **PA16** were grown on LB media overnight. Genomic DNA was extracted according to the manufacturer's protocol (Thermo Scientific GeneJET Genomic DNA Purification Kit). After verification of DNA concentrations using Nanodrop the samples were sequenced using an Illumina platform through a third-party service provider (NovogeneAIT Genomics Singapore). The resulting BAM data was viewed using the Integrative Genomics Viewer [28]. Single Nucleotide Polymorphism (SNP) data were processed via an in-house developed MATLAB application and exported as an Excel spreadsheet file for further analysis.

## CRediT authorship contribution statement

**Nga Yu Poon:** Writing – review & editing, Writing – original draft, Visualization, Validation, Software, Project administration, Methodology, Investigation, Formal analysis, Data curation, Conceptualization. **Anthony J. Sinskey:** Writing – review & editing, Supervision, Conceptualization. **Kang Zhou:** Writing – review & editing, Supervision, Resources, Funding acquisition, Conceptualization.

## Ethical statement

This article does not contain any studies with human participants or animals performed by any of the authors.

## Declaration of competing interest

The author Kang Zhou is an Editorial Board Member for Synthetic and Systems Biotechnology and was not involved in the editorial review or the decision to publish this article. The authors declare that they have no known competing financial interests or personal relationships that could have appeared to influence the work reported in this paper.

## Data Availability

All data supporting this study have been included in this manuscript except the raw RNA sequencing data, which has been submitted to the National Centre of Biotechnology Information Sequence Read Archive (NCBI SRA accession number: PRJNA1158018). Strain **PA16** has been deposited in the DSMZ-German Collection of Microorganisms and Cell Cultures GmbH (DSM 119899).
