# Supplementary material for: Metabolic engineering enables Escherichia coli to grow on 1,3-propanediol
Source: Synth Syst Biotechnol. 2025 Dec 22;12:209–17. doi: 10.1016/j.synbio.2025.11.009 (PMC12796543; doi:10.1016/j.synbio.2025.11.009)
Supplement: Multimedia component 1 [file mmc1.docx]

**Metabolic engineering enables *Escherichia coli* to grow on 1,3-propanediol**

Poon Nga Yu ^1,3^, Anthony J. Sinskey ^2, *^, Kang Zhou ^1,3, *^

^1^ Department of Chemical and Biomolecular Engineering, National University of Singapore, Singapore

^2^ Department of Biology, Massachusetts Institute of Technology, Cambridge, Massachusetts, USA

^3^ Cluster of Food, Chemical and Biotechnology, Singapore Institute of Technology, Singapore

* Corresponding authors: Kang Zhou ([kang.zhou@nus.edu.sg](mailto:kang.zhou@nus.edu.sg)), Anthony J. Sinskey ([asinskey@mit.edu](mailto:asinskey@mit.edu))

# Growth comparison of **PA16** between aerobic and micro aerobic conditions


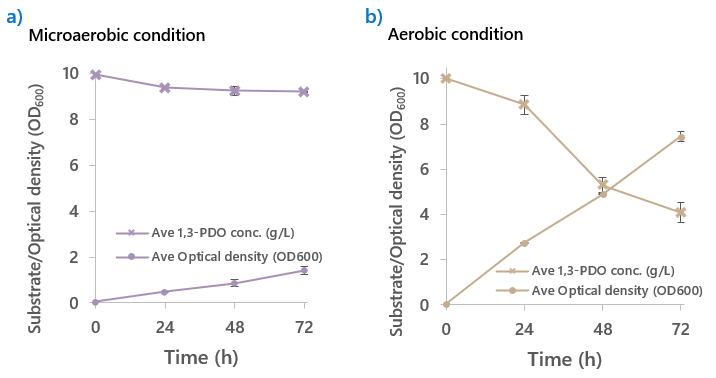


**Figure S1** **Diagrams illustrating the importance of oxygen availability for the 1,3-PDO assimilation pathway**

**(a)** Under limited oxygen conditions, **PA16** exhibited poor growth and limited 1,3-PDO assimilation, reaching an OD_600_ of only 1.4 with 0.8 g/L of 1,3-PDO consumed. **(b)** In contrast, aerobic cultivation restored robust growth, with **PA16** attaining an OD_600_ of 7.44 while consuming 6 g/L of 1,3-PDO.

# Single Nucleotide Polymorphism (SNP) data

## 2.1 SNP data of **BA07Δ** (Parent strain)

| **Index** | **Reference** | **Change** | **Type** | **Gene1** | **Gene 2** |
| --- | --- | --- | --- | --- | --- |
| 366351 | A | T | Intergenic | lacZ | lacI |
| 366352 | C | T | Intergenic | lacZ | lacI |
| 366409 | C | T | Intergenic | lacZ | lacI |
| 491351 | G | C | Intergenic | ybaN | apt |
| 566173 | C | G | Intergenic | intD | renD |
| 566205 | T | C | Intergenic | intD | renD |
| 566245 | G | A | Intergenic | intD | renD |
| 566277 | C | T | Intergenic | intD | renD |
| 566323 | C | T | Intergenic | intD | renD |
| 566326 | T | C | Intergenic | intD | renD |
| 566332 | T | G | Intergenic | intD | renD |
| 566543 | A | T | Intergenic | intD | renD |
| 566547 | C | A | Intergenic | intD | renD |
| 566565 | A | G | Intergenic | intD | renD |
| 566570 | A | C | Intergenic | intD | renD |
| 566597 | T | C | Intergenic | intD | renD |
| 566651 | T | C | Intergenic | intD | renD |
| 566728 | G | T | CDS | renD | NA |
| 566740 | A | G | CDS | renD | NA |
| 566742 | A | C | CDS | renD | NA |
| 578331 | G | T | CDS | rzpD | NA |
| 578427 | G | C | CDS | rzpD | NA |
| 578809 | T | C | CDS | borD | NA |
| 578811 | T | G | CDS | borD | NA |
| 578831 | T | C | CDS | borD | NA |
| 579004 | C | T | Intergenic | borD | ybcV |
| 579127 | T | C | Intergenic | borD | ybcV |
| 579134 | T | C | Intergenic | borD | ybcV |
| 579146 | A | G | Intergenic | borD | ybcV |
| 579271 | G | A | CDS | ybcV | NA |
| 579285 | T | C | CDS | ybcV | NA |
| 579385 | A | G | CDS | ybcV | NA |
| 579457 | A | G | CDS | ybcV | NA |
| 579502 | A | G | CDS | ybcV | NA |
| 579540 | T | C | CDS | ybcV | NA |
| 579651 | C | T | Intergenic | ybcV | ybcW |
| 579671 | T | C | Intergenic | ybcV | ybcW |
| 579717 | A | C | Intergenic | ybcV | ybcW |
| 579811 | C | T | Intergenic | ybcV | ybcW |
| 580020 | G | A | CDS | ybcW | NA |
| 580115 | C | T | Intergenic | ybcW | ylcI |
| 580119 | A | G | Intergenic | ybcW | ylcI |
| 580120 | C | T | Intergenic | ybcW | ylcI |
| 580302 | C | T | CDS | ylcI | NA |
| 580830 | T | A | Intergenic | ylcI | nohD |
| 580831 | G | A | Intergenic | ylcI | nohD |
| 581042 | T | G | CDS | nohD | NA |
| 581056 | A | G | CDS | nohD | NA |
| 581073 | G | A | CDS | nohD | NA |
| 581118 | T | C | CDS | nohD | NA |
| 581127 | G | A | CDS | nohD | NA |
| 581133 | A | G | CDS | nohD | NA |
| 581227 | A | C | CDS | nohD | NA |
| 1011582 | C | T | CDS | uup | NA |
| 1423225 | G | A | CDS | ydaW | NA |
| 1423244 | G | C | CDS | ydaW | NA |
| 1423272 | C | T | CDS | ydaW | NA |
| 1423273 | G | A | CDS | ydaW | NA |
| 1423283 | A | C | CDS | ydaW | NA |
| 1423284 | A | C | CDS | ydaW | NA |
| 1423338 | G | C | Intergenic | ydaW | rzoR |
| 1423404 | G | T | CDS | rzoR | NA |
| 1429021 | T | A | Intergenic | insH5 | stfR |
| 1429022 | T | A | Intergenic | insH5 | stfR |
| 1429024 | A | C | Intergenic | insH5 | stfR |
| 1429027 | C | T | Intergenic | insH5 | stfR |
| 1429069 | T | A | CDS | stfR | NA |
| 1429072 | A | C | CDS | stfR | NA |
| 1429111 | A | C | CDS | stfR | NA |
| 1429114 | C | T | CDS | stfR | NA |
| 1429126 | A | C | CDS | stfR | NA |
| 1429128 | A | G | CDS | stfR | NA |
| 1429160 | C | G | CDS | stfR | NA |
| 1429164 | C | G | CDS | stfR | NA |
| 1429171 | A | G | CDS | stfR | NA |
| 1429204 | C | T | CDS | stfR | NA |
| 1429207 | T | G | CDS | stfR | NA |
| 1429225 | C | T | CDS | stfR | NA |
| 1429228 | T | C | CDS | stfR | NA |
| 1429231 | T | C | CDS | stfR | NA |
| 1429235 | T | C | CDS | stfR | NA |
| 1429236 | T | A | CDS | stfR | NA |
| 1429240 | G | T | CDS | stfR | NA |
| 1429243 | A | C | CDS | stfR | NA |
| 1429246 | A | T | CDS | stfR | NA |
| 1429249 | C | T | CDS | stfR | NA |
| 1429252 | G | A | CDS | stfR | NA |
| 1429255 | G | A | CDS | stfR | NA |
| 1429258 | A | G | CDS | stfR | NA |
| 1429261 | T | C | CDS | stfR | NA |
| 1429273 | T | C | CDS | stfR | NA |
| 1429291 | T | A | CDS | stfR | NA |
| 1429297 | C | G | CDS | stfR | NA |
| 1429300 | T | G | CDS | stfR | NA |
| 1429319 | G | T | CDS | stfR | NA |
| 1429682 | T | G | CDS | stfR | NA |
| 1429692 | T | A | CDS | stfR | NA |
| 1429702 | A | C | CDS | stfR | NA |
| 1429708 | A | G | CDS | stfR | NA |
| 1429714 | A | C | CDS | stfR | NA |
| 1429724 | A | G | CDS | stfR | NA |
| 1429732 | G | A | CDS | stfR | NA |
| 1429735 | A | G | CDS | stfR | NA |
| 1429741 | A | G | CDS | stfR | NA |
| 1429744 | T | C | CDS | stfR | NA |
| 1429747 | G | C | CDS | stfR | NA |
| 1429750 | C | T | CDS | stfR | NA |
| 1429753 | G | A | CDS | stfR | NA |
| 1429756 | C | A | CDS | stfR | NA |
| 1429759 | G | A | CDS | stfR | NA |
| 1429767 | C | T | CDS | stfR | NA |
| 1429780 | A | G | CDS | stfR | NA |
| 1429783 | G | A | CDS | stfR | NA |
| 1429816 | C | T | CDS | stfR | NA |
| 1429820 | A | G | CDS | stfR | NA |
| 1429823 | A | C | CDS | stfR | NA |
| 1636068 | T | C | CDS | nohA | NA |
| 1636074 | C | T | CDS | nohA | NA |
| 1636128 | C | T | CDS | nohA | NA |
| 1636145 | T | C | CDS | nohA | NA |
| 1636173 | T | C | CDS | nohA | NA |
| 1636382 | C | A | Intergenic | nohA | ynfO |
| 1636455 | G | A | Intergenic | nohA | ynfO |
| 1636484 | C | T | Intergenic | nohA | ynfO |
| 1636503 | T | C | Intergenic | nohA | ynfO |
| 2895352 | A | G | CDS | ygcQ | NA |
| 3118136 | G | A | CDS | yghJ | NA |
| 1429682 | T | G | CDS | stfR | NA |
| 1429692 | T | A | CDS | stfR | NA |
| 1429702 | A | C | CDS | stfR | NA |
| 1429708 | A | G | CDS | stfR | NA |
| 1429714 | A | C | CDS | stfR | NA |
| 1429724 | A | G | CDS | stfR | NA |
| 1429732 | G | A | CDS | stfR | NA |
| 1429735 | A | G | CDS | stfR | NA |
| 1429741 | A | G | CDS | stfR | NA |
| 1429744 | T | C | CDS | stfR | NA |
| 1429747 | G | C | CDS | stfR | NA |
| 1429750 | C | T | CDS | stfR | NA |
| 1429753 | G | A | CDS | stfR | NA |
| 1429756 | C | A | CDS | stfR | NA |
| 1429759 | G | A | CDS | stfR | NA |
| 1429767 | C | T | CDS | stfR | NA |
| 1429780 | A | G | CDS | stfR | NA |
| 1429783 | G | A | CDS | stfR | NA |
| 1429816 | C | T | CDS | stfR | NA |
| 1429820 | A | G | CDS | stfR | NA |
| 1429823 | A | C | CDS | stfR | NA |
| 1636068 | T | C | CDS | nohA | NA |
| 1636074 | C | T | CDS | nohA | NA |
| 1636128 | C | T | CDS | nohA | NA |
| 1636145 | T | C | CDS | nohA | NA |
| 1636382 | C | A | Intergenic | nohA | ynfO |
| 1636484 | C | T | Intergenic | nohA | ynfO |
| 1636503 | T | C | Intergenic | nohA | ynfO |
| 2339589 | T | A | CDS | ubiG | NA |
| 2895352 | A | G | CDS | ygcQ | NA |
| 3118136 | G | A | CDS | yghJ | NA |
| 3326547 | C | T | CDS | ftsH | NA |

## 2.2 SNP data of **PA1**

| **Index** | **Reference** | **Change** | **Type** | **Gene1** | **Gene 2** |
| --- | --- | --- | --- | --- | --- |
| 158868 | C | G | CDS | pcnB | NA |
| 257908 | G | A | CDS | crl | NA |
| 366351 | A | T | Intergenic | lacZ | lacI |
| 366352 | C | T | Intergenic | lacZ | lacI |
| 366409 | C | T | Intergenic | lacZ | lacI |
| 366710 | A | G | CDS | lacI | NA |
| 491351 | G | C | Intergenic | ybaN | apt |
| 566173 | C | G | Intergenic | intD | renD |
| 566205 | T | C | Intergenic | intD | renD |
| 566245 | G | A | Intergenic | intD | renD |
| 566277 | C | T | Intergenic | intD | renD |
| 566323 | C | T | Intergenic | intD | renD |
| 566326 | T | C | Intergenic | intD | renD |
| 566332 | T | G | Intergenic | intD | renD |
| 566356 | T | C | Intergenic | intD | renD |
| 566565 | A | G | Intergenic | intD | renD |
| 566570 | A | C | Intergenic | intD | renD |
| 566597 | T | C | Intergenic | intD | renD |
| 566651 | T | C | Intergenic | intD | renD |
| 566728 | G | T | CDS | renD | NA |
| 566740 | A | G | CDS | renD | NA |
| 566742 | A | C | CDS | renD | NA |
| 578331 | G | T | CDS | rzpD | NA |
| 578427 | G | C | CDS | rzpD | NA |
| 578809 | T | C | CDS | borD | NA |
| 578811 | T | G | CDS | borD | NA |
| 578831 | T | C | CDS | borD | NA |
| 579004 | C | T | Intergenic | borD | ybcV |
| 579127 | T | C | Intergenic | borD | ybcV |
| 579134 | T | C | Intergenic | borD | ybcV |
| 579146 | A | G | Intergenic | borD | ybcV |
| 579271 | G | A | CDS | ybcV | NA |
| 579285 | T | C | CDS | ybcV | NA |
| 579385 | A | G | CDS | ybcV | NA |
| 579457 | A | G | CDS | ybcV | NA |
| 579502 | A | G | CDS | ybcV | NA |
| 579540 | T | C | CDS | ybcV | NA |
| 579651 | C | T | Intergenic | ybcV | ybcW |
| 579671 | T | C | Intergenic | ybcV | ybcW |
| 579717 | A | C | Intergenic | ybcV | ybcW |
| 579811 | C | T | Intergenic | ybcV | ybcW |
| 580020 | G | A | CDS | ybcW | NA |
| 580115 | C | T | Intergenic | ybcW | ylcI |
| 580119 | A | G | Intergenic | ybcW | ylcI |
| 580120 | C | T | Intergenic | ybcW | ylcI |
| 580302 | C | T | CDS | ylcI | NA |
| 580830 | T | A | Intergenic | ylcI | nohD |
| 580831 | G | A | Intergenic | ylcI | nohD |
| 581042 | T | G | CDS | nohD | NA |
| 581056 | A | G | CDS | nohD | NA |
| 581073 | G | A | CDS | nohD | NA |
| 581118 | T | C | CDS | nohD | NA |
| 581127 | G | A | CDS | nohD | NA |
| 581133 | A | G | CDS | nohD | NA |
| 581227 | A | C | CDS | nohD | NA |
| 1011582 | C | T | CDS | uup | NA |
| 1423225 | G | A | CDS | ydaW | NA |
| 1423244 | G | C | CDS | ydaW | NA |
| 1423272 | C | T | CDS | ydaW | NA |
| 1423273 | G | A | CDS | ydaW | NA |
| 1423283 | A | C | CDS | ydaW | NA |
| 1423284 | A | C | CDS | ydaW | NA |
| 1423338 | G | C | Intergenic | ydaW | rzoR |
| 1428945 | C | T | CDS | lomR | NA |
| 1428954 | T | A | CDS | lomR | NA |
| 1428963 | G | T | CDS | lomR | NA |
| 1428966 | T | G | CDS | lomR | NA |
| 1429021 | T | A | Intergenic | insH5 | stfR |
| 1429022 | T | A | Intergenic | insH5 | stfR |
| 1429024 | A | C | Intergenic | insH5 | stfR |
| 1429027 | C | T | Intergenic | insH5 | stfR |
| 1429069 | T | A | CDS | stfR | NA |
| 1429072 | A | C | CDS | stfR | NA |
| 1429111 | A | C | CDS | stfR | NA |
| 1429114 | C | T | CDS | stfR | NA |
| 1429126 | A | C | CDS | stfR | NA |
| 1429128 | A | G | CDS | stfR | NA |
| 1429160 | C | G | CDS | stfR | NA |
| 1429164 | C | G | CDS | stfR | NA |
| 1429171 | A | G | CDS | stfR | NA |
| 1429204 | C | T | CDS | stfR | NA |
| 1429207 | T | G | CDS | stfR | NA |
| 1429225 | C | T | CDS | stfR | NA |
| 1429228 | T | C | CDS | stfR | NA |
| 1429231 | T | C | CDS | stfR | NA |
| 1429235 | T | C | CDS | stfR | NA |
| 1429236 | T | A | CDS | stfR | NA |
| 1429240 | G | T | CDS | stfR | NA |
| 1429243 | A | C | CDS | stfR | NA |
| 1429246 | A | T | CDS | stfR | NA |
| 1429249 | C | T | CDS | stfR | NA |
| 1429252 | G | A | CDS | stfR | NA |
| 1429255 | G | A | CDS | stfR | NA |
| 1429258 | A | G | CDS | stfR | NA |
| 1429261 | T | C | CDS | stfR | NA |
| 1429273 | T | C | CDS | stfR | NA |
| 1429291 | T | A | CDS | stfR | NA |
| 1429297 | C | G | CDS | stfR | NA |
| 1429300 | T | G | CDS | stfR | NA |
| 1429319 | G | T | CDS | stfR | NA |
| 1429345 | T | G | CDS | stfR | NA |
| 1429353 | C | T | CDS | stfR | NA |
| 1429354 | A | G | CDS | stfR | NA |
| 1429360 | C | T | CDS | stfR | NA |
| 1429364 | T | C | CDS | stfR | NA |
| 1429404 | C | T | CDS | stfR | NA |
| 1429416 | A | G | CDS | stfR | NA |
| 1429417 | C | T | CDS | stfR | NA |
| 1429425 | C | A | CDS | stfR | NA |
| 1429435 | G | A | CDS | stfR | NA |
| 1429442 | A | G | CDS | stfR | NA |
| 1429444 | T | C | CDS | stfR | NA |
| 1429453 | C | T | CDS | stfR | NA |
| 1429454 | A | G | CDS | stfR | NA |
| 1429479 | A | T | CDS | stfR | NA |
| 1429482 | C | T | CDS | stfR | NA |
| 1429484 | G | A | CDS | stfR | NA |
| 1429492 | T | A | CDS | stfR | NA |
| 1429493 | G | A | CDS | stfR | NA |
| 1429495 | G | T | CDS | stfR | NA |
| 1429510 | A | C | CDS | stfR | NA |
| 1429522 | A | C | CDS | stfR | NA |
| 1429534 | C | T | CDS | stfR | NA |
| 1429537 | G | A | CDS | stfR | NA |
| 1429543 | G | A | CDS | stfR | NA |
| 1429550 | T | G | CDS | stfR | NA |
| 1429551 | C | A | CDS | stfR | NA |
| 1429558 | T | C | CDS | stfR | NA |
| 1429562 | A | G | CDS | stfR | NA |
| 1429569 | G | A | CDS | stfR | NA |
| 1429571 | A | G | CDS | stfR | NA |
| 1429580 | A | G | CDS | stfR | NA |
| 1429609 | T | C | CDS | stfR | NA |
| 1429612 | C | A | CDS | stfR | NA |
| 1429615 | T | C | CDS | stfR | NA |
| 1429632 | G | A | CDS | stfR | NA |
| 1429682 | T | G | CDS | stfR | NA |
| 1429692 | T | A | CDS | stfR | NA |
| 1429702 | A | C | CDS | stfR | NA |
| 1429708 | A | G | CDS | stfR | NA |
| 1429714 | A | C | CDS | stfR | NA |
| 1429724 | A | G | CDS | stfR | NA |
| 1429732 | G | A | CDS | stfR | NA |
| 1429735 | A | G | CDS | stfR | NA |
| 1429741 | A | G | CDS | stfR | NA |
| 1429744 | T | C | CDS | stfR | NA |
| 1429747 | G | C | CDS | stfR | NA |
| 1429750 | C | T | CDS | stfR | NA |
| 1429753 | G | A | CDS | stfR | NA |
| 1429756 | C | A | CDS | stfR | NA |
| 1429759 | G | A | CDS | stfR | NA |
| 1429767 | C | T | CDS | stfR | NA |
| 1429780 | A | G | CDS | stfR | NA |
| 1429783 | G | A | CDS | stfR | NA |
| 1429816 | C | T | CDS | stfR | NA |
| 1429820 | A | G | CDS | stfR | NA |
| 1429823 | A | C | CDS | stfR | NA |
| 1636068 | T | C | CDS | nohA | NA |
| 1636074 | C | T | CDS | nohA | NA |
| 1636173 | T | C | CDS | nohA | NA |
| 1636455 | G | A | Intergenic | nohA | ynfO |
| 1636484 | C | T | Intergenic | nohA | ynfO |
| 1636503 | T | C | Intergenic | nohA | ynfO |
| 2339589 | T | A | CDS | ubiG | NA |
| 2895352 | A | G | CDS | ygcQ | NA |
| 3118136 | G | A | CDS | yghJ | NA |
| 3326547 | C | T | CDS | ftsH | NA |

## 2.3 SNP data of **PA16**

| **Index** | **Reference** | **Change** | **Type** | **Gene1** | **Gene 2** |
| --- | --- | --- | --- | --- | --- |
| 158868 | C | G | CDS | pcnB | NA |
| 158934 | C | G | CDS | pcnB | NA |
| 366351 | A | T | Intergenic | lacZ | lacI |
| 366352 | C | T | Intergenic | lacZ | lacI |
| 366409 | C | T | Intergenic | lacZ | lacI |
| 366710 | A | G | CDS | lacI | NA |
| 491351 | G | C | Intergenic | ybaN | apt |
| 566173 | C | G | Intergenic | intD | renD |
| 566205 | T | C | Intergenic | intD | renD |
| 566245 | G | A | Intergenic | intD | renD |
| 566277 | C | T | Intergenic | intD | renD |
| 566323 | C | T | Intergenic | intD | renD |
| 566326 | T | C | Intergenic | intD | renD |
| 566332 | T | G | Intergenic | intD | renD |
| 566356 | T | C | Intergenic | intD | renD |
| 566597 | T | C | Intergenic | intD | renD |
| 566651 | T | C | Intergenic | intD | renD |
| 566728 | G | T | CDS | renD | NA |
| 566740 | A | G | CDS | renD | NA |
| 566742 | A | C | CDS | renD | NA |
| 578427 | G | C | CDS | rzpD | NA |
| 578809 | T | C | CDS | borD | NA |
| 578811 | T | G | CDS | borD | NA |
| 578831 | T | C | CDS | borD | NA |
| 579004 | C | T | Intergenic | borD | ybcV |
| 579127 | T | C | Intergenic | borD | ybcV |
| 579134 | T | C | Intergenic | borD | ybcV |
| 579146 | A | G | Intergenic | borD | ybcV |
| 579271 | G | A | CDS | ybcV | NA |
| 579285 | T | C | CDS | ybcV | NA |
| 579385 | A | G | CDS | ybcV | NA |
| 579457 | A | G | CDS | ybcV | NA |
| 579502 | A | G | CDS | ybcV | NA |
| 579540 | T | C | CDS | ybcV | NA |
| 579651 | C | T | Intergenic | ybcV | ybcW |
| 579671 | T | C | Intergenic | ybcV | ybcW |
| 579717 | A | C | Intergenic | ybcV | ybcW |
| 579811 | C | T | Intergenic | ybcV | ybcW |
| 580020 | G | A | CDS | ybcW | NA |
| 580115 | C | T | Intergenic | ybcW | ylcI |
| 580119 | A | G | Intergenic | ybcW | ylcI |
| 580120 | C | T | Intergenic | ybcW | ylcI |
| 580302 | C | T | CDS | ylcI | NA |
| 581042 | T | G | CDS | nohD | NA |
| 581056 | A | G | CDS | nohD | NA |
| 581073 | G | A | CDS | nohD | NA |
| 581127 | G | A | CDS | nohD | NA |
| 581133 | A | G | CDS | nohD | NA |
| 581227 | A | C | CDS | nohD | NA |
| 1011582 | C | T | CDS | uup | NA |
| 1423225 | G | A | CDS | ydaW | NA |
| 1423244 | G | C | CDS | ydaW | NA |
| 1423272 | C | T | CDS | ydaW | NA |
| 1423273 | G | A | CDS | ydaW | NA |
| 1423283 | A | C | CDS | ydaW | NA |
| 1423284 | A | C | CDS | ydaW | NA |
| 1423338 | G | C | Intergenic | ydaW | rzoR |
| 1423404 | G | T | CDS | rzoR | NA |
| 1429021 | T | A | Intergenic | insH5 | stfR |
| 1429022 | T | A | Intergenic | insH5 | stfR |
| 1429024 | A | C | Intergenic | insH5 | stfR |
| 1429027 | C | T | Intergenic | insH5 | stfR |
| 1429069 | T | A | CDS | stfR | NA |
| 1429072 | A | C | CDS | stfR | NA |
| 1429111 | A | C | CDS | stfR | NA |
| 1429114 | C | T | CDS | stfR | NA |
| 1429126 | A | C | CDS | stfR | NA |
| 1429128 | A | G | CDS | stfR | NA |
| 1429160 | C | G | CDS | stfR | NA |
| 1429164 | C | G | CDS | stfR | NA |
| 1429171 | A | G | CDS | stfR | NA |
| 1429204 | C | T | CDS | stfR | NA |
| 1429207 | T | G | CDS | stfR | NA |
| 1429225 | C | T | CDS | stfR | NA |
| 1429228 | T | C | CDS | stfR | NA |
| 1429231 | T | C | CDS | stfR | NA |
| 1429235 | T | C | CDS | stfR | NA |
| 1429236 | T | A | CDS | stfR | NA |
| 1429240 | G | T | CDS | stfR | NA |
| 1429243 | A | C | CDS | stfR | NA |
| 1429246 | A | T | CDS | stfR | NA |
| 1429249 | C | T | CDS | stfR | NA |
| 1429252 | G | A | CDS | stfR | NA |
| 1429255 | G | A | CDS | stfR | NA |
| 1429258 | A | G | CDS | stfR | NA |
| 1429261 | T | C | CDS | stfR | NA |
| 1429273 | T | C | CDS | stfR | NA |
| 1429291 | T | A | CDS | stfR | NA |
| 1429297 | C | G | CDS | stfR | NA |
| 1429300 | T | G | CDS | stfR | NA |
| 1429319 | G | T | CDS | stfR | NA |
| 1429345 | T | G | CDS | stfR | NA |
| 1429353 | C | T | CDS | stfR | NA |
| 1429354 | A | G | CDS | stfR | NA |
| 1429360 | C | T | CDS | stfR | NA |
| 1429364 | T | C | CDS | stfR | NA |
| 1429404 | C | T | CDS | stfR | NA |
| 1429416 | A | G | CDS | stfR | NA |
| 1429417 | C | T | CDS | stfR | NA |
| 1429425 | C | A | CDS | stfR | NA |
| 1429435 | G | A | CDS | stfR | NA |
| 1429442 | A | G | CDS | stfR | NA |
| 1429444 | T | C | CDS | stfR | NA |
| 1429453 | C | T | CDS | stfR | NA |
| 1429454 | A | G | CDS | stfR | NA |
| 1429479 | A | T | CDS | stfR | NA |
| 1429482 | C | T | CDS | stfR | NA |
| 1429484 | G | A | CDS | stfR | NA |
| 1429492 | T | A | CDS | stfR | NA |
| 1429493 | G | A | CDS | stfR | NA |
| 1429495 | G | T | CDS | stfR | NA |
| 1429510 | A | C | CDS | stfR | NA |
| 1429522 | A | C | CDS | stfR | NA |
| 1429534 | C | T | CDS | stfR | NA |
| 1429537 | G | A | CDS | stfR | NA |
| 1429543 | G | A | CDS | stfR | NA |
| 1429550 | T | G | CDS | stfR | NA |
| 1429551 | C | A | CDS | stfR | NA |
| 1429558 | T | C | CDS | stfR | NA |
| 1429562 | A | G | CDS | stfR | NA |
| 1429569 | G | A | CDS | stfR | NA |
| 1429571 | A | G | CDS | stfR | NA |
| 1429580 | A | G | CDS | stfR | NA |
| 1429609 | T | C | CDS | stfR | NA |
| 1429612 | C | A | CDS | stfR | NA |
| 1429615 | T | C | CDS | stfR | NA |
| 1429632 | G | A | CDS | stfR | NA |
| 1429682 | T | G | CDS | stfR | NA |
| 1429692 | T | A | CDS | stfR | NA |
| 1429702 | A | C | CDS | stfR | NA |
| 1429708 | A | G | CDS | stfR | NA |
| 1429714 | A | C | CDS | stfR | NA |
| 1429724 | A | G | CDS | stfR | NA |
| 1429732 | G | A | CDS | stfR | NA |
| 1429735 | A | G | CDS | stfR | NA |
| 1429741 | A | G | CDS | stfR | NA |
| 1429744 | T | C | CDS | stfR | NA |
| 1429747 | G | C | CDS | stfR | NA |
| 1429750 | C | T | CDS | stfR | NA |
| 1429753 | G | A | CDS | stfR | NA |
| 1429756 | C | A | CDS | stfR | NA |
| 1429759 | G | A | CDS | stfR | NA |
| 1429767 | C | T | CDS | stfR | NA |
| 1429780 | A | G | CDS | stfR | NA |
| 1429783 | G | A | CDS | stfR | NA |
| 1429816 | C | T | CDS | stfR | NA |
| 1429820 | A | G | CDS | stfR | NA |
| 1429823 | A | C | CDS | stfR | NA |
| 1636068 | T | C | CDS | nohA | NA |
| 1636074 | C | T | CDS | nohA | NA |
| 1636128 | C | T | CDS | nohA | NA |
| 1636145 | T | C | CDS | nohA | NA |
| 1636382 | C | A | Intergenic | nohA | ynfO |
| 1636484 | C | T | Intergenic | nohA | ynfO |
| 1636503 | T | C | Intergenic | nohA | ynfO |
| 2339589 | T | A | CDS | ubiG | NA |
| 2895352 | A | G | CDS | ygcQ | NA |
| 3118136 | G | A | CDS | yghJ | NA |
| 3326547 | C | T | CDS | ftsH | NA |
